# PE and PET oligomers’ interplay with membrane bilayers

**DOI:** 10.1038/s41598-022-06217-4

**Published:** 2022-02-09

**Authors:** Joni Järvenpää, Milla Perkkiö, Riikka Laitinen, Maija Lahtela-Kakkonen

**Affiliations:** School of Pharmacy, Faculty of Health Sciences, University of Eastern Finland, 70210 Kuopio, Finland

**Keywords:** Computational chemistry, Molecular dynamics, Membrane lipids, Polymer chemistry, Materials chemistry, Environmental sciences

## Abstract

The prevalence of microplastic pollution in nature and foodstuffs is fairly well identified. However, studies of micro- or nanoplastics’ cell membrane permeation and health effects in humans are lacking. Our study focuses on examining the interactions of polyethylene (PE) and polyethylene terephthalate (PET) with bilayer membranes. We have performed molecular dynamics simulations to study how plastic oligomers behave in bilayers. In addition, we have studied membrane permeation of PE and Bis(2-hydroxyethyl) terephthalate (BHET), a type of PET monomer, with Parallel Artificial Membrane Permeability Assay (PAMPA). As a result, in simulations the molecules exhibited different movements and preferred locations in membrane. PAMPA studies suggested similar preferences in membrane, especially for PE plastic. Our results suggest that passive diffusion could be an important transport mechanism into cells for some small plastic oligomers. Both molecular dynamics simulations and PAMPA have potential for micro- and nanoplastics research.

## Introduction

Most plastics are inert materials, but they may become more problematic when they break down into less than 5 mm sized microplastics (MPs). Due to the large surface area, MPs can better absorb environmental pollutants and transfer them, therefore making them potentially much more dangerous than larger pieces of plastics^1,2^. Plastics smaller than 1 µm or 100 nm can be classified as nanoplastics. There is a decent amount of research about the prevalence and identification of MPs in nature, marine life and foodstuffs, although the research suffers from a lack of standardization^3–5^. There is, however, a significant lack of research of MP’s health effects on humans and a deeper understanding into the mechanisms of MP transport is needed. Whether certain plastics could be more dangerous than others is also not known.

Some in vitro studies show that microplastics can enter cells in small quantities without clear negative effects, although evidence is still very limited, and the mechanisms of transport are not clear^6–8^. The possible mechanisms could be passive diffusion or endocytosis but few testing methods have been developed for studying the transport in microplastic research.

Molecular dynamics (MD) can be applied to observe dynamic behavior of molecules and study molecular level interactions between, for example, small molecule-lipid, lipid-lipid or macromolecule-lipid^9,10^. MD has been used in the study of membrane permeation of many different compounds. There have been very few studies about permeation of MPs or associated compounds, although MD has been used to study various other effects of microplastics, such as absorption of pollutants or its effects on proteins^11–13^.

Parallel Artificial Membrane Permeability Assay (PAMPA) can help predict passive transcellular permeation of small molecules through artificial membranes^14^. Passive permeation in various types of biomembranes, such as intestines, skin or blood–brain-barrier can be studied using different types of membranes, lipids, solubilizing agents, solvents and analysis methods, allowing flexibility and cost efficiency for studying^15,16^. Typically PAMPA has been used for research into passive absorption of drugs^17,18^. To our knowledge, PAMPA has not been used in the study of microplastics before.

Our study focuses on examining the transport of polyethylene (PE) and polyethylene terephthalate (PET) across bilayer membranes which mimic cell membranes. We have performed molecular dynamics simulations to study the movement of plastic monomers and tetramers in lipid bilayers. In addition, we have explored experimentally the permeation of PE and Bis(2-hydroxyethyl) terephthalate (BHET) with PAMPA. Our results indicate that passive diffusion could potentially be an important transport mechanism into cells for some small plastic oligomers.

## Materials and methods

In MD simulations PE and PET, two structurally different common plastics, were used as monomers and tetramers. BHET was used as the PET monomer in both simulations and PAMPA, as the monoester 2-hydroxyethyl terephthalic acid was considered too hydrophilic. Ethanol was used as a reference molecule as it can easily diffuse through cell membranes^19,20^. The small molecule structure files were generated using CHARMM-GUI Ligand Reader and Modeler^21^. Parameters for the molecules were prepared using The CHARMM General Force Field program^22,23^.

The bilayer-small-molecule -systems were constructed using CHARMM-GUI Membrane-Builder^24^. Homogenous bilayers with a total of 128 lipids were constructed with 64 for each side of either Dipalmitoylphosphatidylcholine (DPPC) or 1-Palmitoyl-2-oleoylphosphatidylcholine (POPC). A layer (40 Å thickness) of TIP3 water model was added on either side of the bilayer, except with PET tetramer where 50 Å was used. Two starting locations were applied for the monomers and tetramers (1) at the centers of the bilayers, (2) translated into the water phase. A translation of 40 Å was used for most molecules, while 50 Å was used for the PET tetramer, because it is significantly larger. All studied molecules were oriented with the principal axis along the z-coordinate. All water molecules within 2 Å distance from the studied molecules were removed using the VMD molecular graphics viewer (version 1.9.3) in order to avoid clashes at the beginning of minimization and equilibration runs^25^. Pure DPPC and POPC bilayers and water without other added molecules were also simulated as a reference. Simulation set-up is summarized in Table 1.

**Table 1:** Molecular dynamics simulation system set-up.

Bilayer Lipid 1	DPPC										
Added molecule	PE monomer		PE tetramer		PET monomer		PET tetramer		EtOH		Pure lipid
Location of molecule at simulation start relative to bilayer	IN	OUT	IN	OUT	IN	OUT	IN	OUT	IN	OUT	
Number 100 ns simulation runs	3	3	3	3	3	3	3	3	3	3	3

Bilayer Lipid 2	POPC										
Added molecule	PE monomer		PE tetramer		PET monomer		PET tetramer		EtOH		Pure lipid
Location of molecule at simulation start relative to bilayer	IN	OUT	IN	OUT	IN	OUT	IN	OUT	IN	OUT	
Number 100 ns simulation runs	3	3	3	3	3	3	3	3	3	3	3

Simulations were performed with Gromacs version 2019.4^26^. CHARMM36m force field was applied in simulations. The system was equilibrated for 1 ns in NVT ensemble and after that 1 ns in NPT. Restraints of 1000 units kJ/mol nm^2^ in X, Y, Z directions were used on the small molecules during the equilibration runs. NPT ensemble was used for production runs with Nose–Hoover thermostat on selected temperature and Parrinello-Rahman barostat for the pressure. Temperature of 323 K was used for DPPC and 310 K for POPC in all runs. The temperature for DPPC was set higher due to the pure DPPC being in gel phase at around human body temperature^27^. Production runs of 100 ns were used all with three replications. Various Gromacs tools were used for the analysis of the results. The thickness of the membrane was calculated as the median distance of phosphorous atom density peaks of bilayer leaflets. The area per lipid was calculated by dividing the XY-surface plane area (in nm^2^) by the number of lipids in one leaflet.

Experimental membrane permeation. In PAMPA experiments the membranes were prepared by adding 15 µl of 10% (m/V) l-α-phosphatidylcholine in dodecane into Durapore PVDF (polyvinylidene fluoride) membrane filters (Merc Millipore Ltd. Ireland) with pore sizes of 0.1 µm and 0.22 µm. The moistened membranes were immediately placed vertically between two 3 ml volume side-by-side chambers with an effective permeation area of 0.64 cm^2^. 20 mg of samples of PE (Sigma Aldrich, fine powder, 40–48 µm particle size) or 30 mg of BHET (Sigma Aldrich, large flakes) in 3 ml of water were added into the donor chambers which were set at 37 °C with a water bath (M3, Lauda, Köningshofen, Germany) and mixed with magnetic mixers (PermeGear Inc., Hellertown, PA, USA). 2 ml samples were taken (and immediately replaced with water) from the acceptor chambers at 6, 24, 48 and 72 h time points for PE, and with 1, 6 and 24 h time points for BHET. All experiments were done in triplicate. The one-tailed Welch’s t-test was used for determining whether the larger membrane pore sizes in PAMPA caused significant rises in plastic concentrations. Values were considered significantly different when P < 0.05.

PAMPA samples were analyzed using ^1^H NMR (Bruker Avance III HD 600 MHz NMR system). The PE samples were 500 µl of PAMPA sample with 25 µl 2 mM TSP (3-(Trimethylsilyl)propanoic acid) in D_2_O (deuterated water) added. BHET samples were first evaporated and then dissolved in 650 µl deuterated chloroform with 0.03% (m/V) TMS (Tetramethylsilane) and 5% TFA (trifluoroacetic acid) was added to increase solubility. The NMR peak integrals of the largest and most distinct peaks were used for concentration comparisons, the aromatic hydrogen peak was used for BHET and the methylene hydrogens for PE (Fig. S1). As the study arrangements were different for PE and BHET the concentration differences cannot be directly compared to each other.

## Results and discussion

MD simulations were performed for PE and PET monomers or tetramers with lipid only and lipid with ethanol as references (Table 1). Each simulation was 100 ns and three replicates were performed for runs. There were two possible starting configurations of system: (1) molecule was located in water or (2) molecule was inside the membrane. All simulations were run with both POPC and DPPC membranes. Both membrane types exhibited very similar results, and for clarity, only differences are stated in text.

Analysis of membrane. Membranes were evaluated by calculating the surface area per lipid molecule which was found to not fluctuate significantly during simulations. The area varied between 0.601 and 0.623 nm^2^ for PE and PET in DPPC, and 0.627–0.663 nm^2^ in POPC membranes. Reference pure DPPC membrane had an average area of 0.601 nm^2^ and pure POPC had 0.652 nm^2^. Ethanol caused an average area of 0.635 nm^2^ in DPPC, and 0.658 nm^2^ in POPC membranes. There was no effect based on the movement of PE or PET in any simulated systems. There were only small differences (0–5%) between the areas at the beginning of the simulations originating likely from preprocessing. Similarly, the thickness of the membrane was not fluctuated by molecules entering the membrane. For PE and PET the thickness varied between 3.91–4.00 nm in DPPC membranes, and 3.79–3.98 nm in POPC membranes. Reference thickness for pure DPPC was 4.04 nm and 3.86 nm for POPC membranes. Ethanol caused an average thickness of 3.87 nm in DPPC, and 3.84 nm in POPC membranes. Some fluctuation was observed in replicates, but no trend was visible. Some of the variations were likely caused due to the starting box size settling differently during equilibration runs. Also, possible small effects caused by the single studied molecules would have been drowned out by the large amounts of lipids. The variations in thickness between runs were tested by generating multiple systems from scratch and running equilibration runs on them. The differences in membrane thickness between different tests of the same set-up (i.e. PET-monomer outside POPC membrane) was similar in size as between different set-ups (i.e. PET vs PE simulations) and was seen to originate from system settling which was not affected by the added molecules. Based on our findings the area or thickness of the membrane was not found to be affected by the molecules. Although this does not refute the possibility, as the molecule concentration was very small. With cholesterol, the effects on membrane structure are visible only at higher concentrations and plastics could only have visible effects similarly at higher concentrations^28^.

Additionally, the order parameters for the lipids’ acyl chains were calculated for the DPPC and POPC bilayers. The order parameters were well in compliance with previous studies on the lipids with no significant variation found caused by the added molecules^29^. The single added molecules compared to the large amount of lipids may explain why discernible differences between simulations were not found.

**Figure 1:**
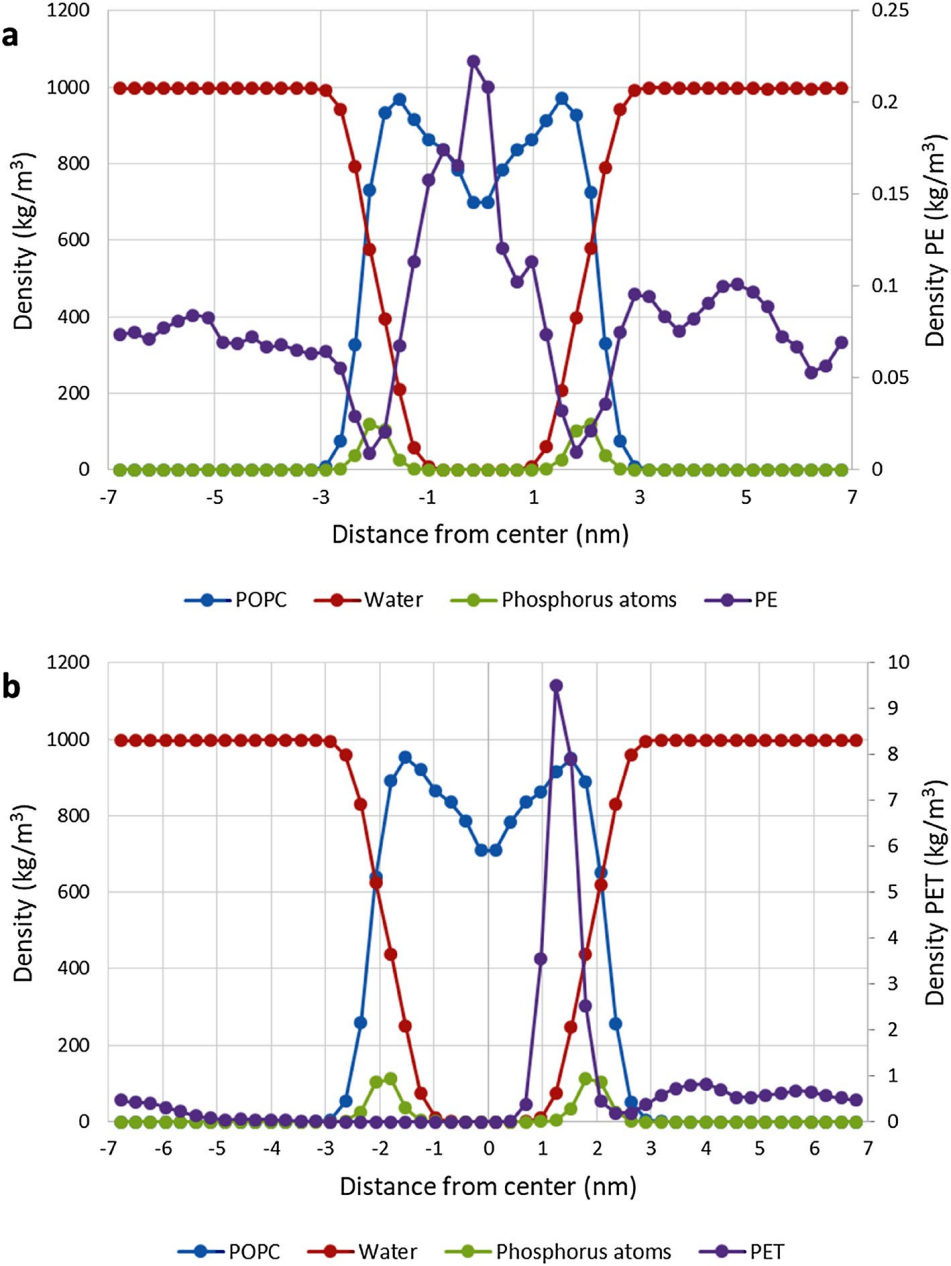
Mass density profiles. (**a**): The mass density profile for PE monomer simulation outside POPC membrane shows that PE heavily prefers to locate in the center of membrane with some movement in water phase. (**b**): The mass density profile for PET monomer shows that PET heavily prefers to locate in the headgroups region of membrane.

**Figure 2:**
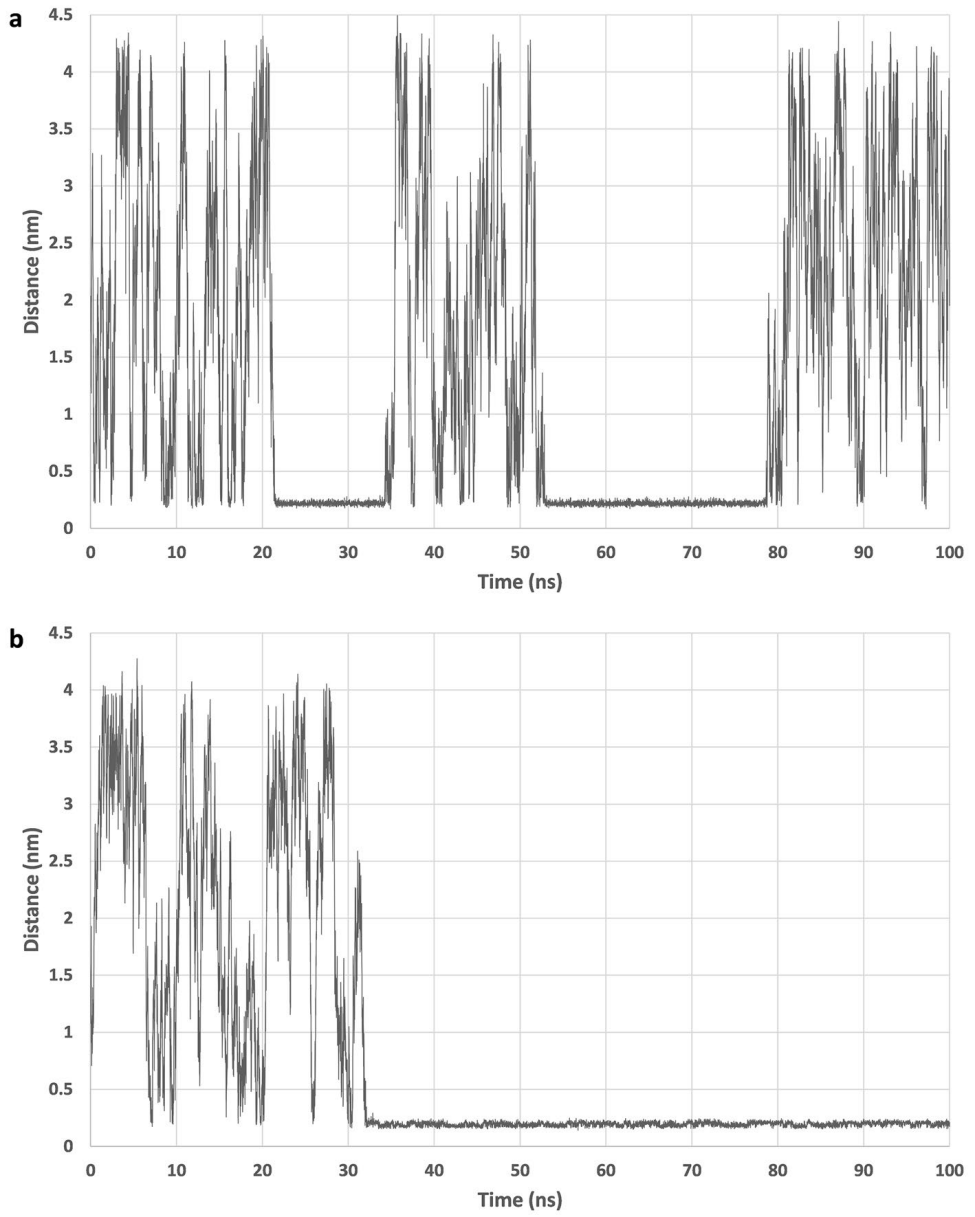
Distance from membrane. (**a**): The distance of PE monomer from the POPC membrane atoms plotted with respect to simulation time in a single selected example simulation run. Figure shows random movement in water at first until at 22 ns the molecule moved inside the membrane, exited at 35 ns and did another enter and exit between 52 and 78 ns. (**b**): PET monomer showed random movement in water at first but entered the membrane at 30 ns and remained there until the end of the simulation.

The mass density profiles (Figs. 1, S2) for the different molecules in the system were also used to validate that the membrane retained its structure during simulation. The density profiles also provided information about those regions of the system which small molecules could prefer (Figs. 2, S3). The profiles showed clear preferenced locations for the different molecules with parallel simulations showing only minor variance. PET monomer preferred a location at the lipid headgroups at around ± 1.25 nm from the bilayer center. Both PE monomer and tetramer preferred the center of the membrane with the lowest density close to the phosphate density peak, almost at the same location as the PET monomer density peak. Reference molecule ethanol preferred the same location as the PET monomer although with more movement in water phase.

Dynamics of PE and PET was analyzed primarily with a combination of four tools: (1) visual inspection of the trajectories, (2) the density graphs and membrane thickness, (3) the distances of the small molecules from the lipid molecules over time and root mean square deviations (RMSD) of the small molecules over time. The lipid head groups region was considered to be roughly ± 1.0–2.5 nm distance from bilayer center and the lipid tails region ± 1.0 nm distance from center.

By visual inspection PE monomer moved freely inside the membrane traversing all parts of it. It was easily able to exit or enter the membrane, but heavily preferred to stay inside, especially in the center and inside the hydrophobic lipid tails region (Figs. 1a, 2a). Ethanol, the reference molecule, behaved most similarly to the PE monomer moving quite rapidly and easily between water and bilayer. Ethanol, however, preferred the water phase and the headgroups region while mostly avoiding the lipid tails region and moved slightly slower inside the membrane compared to PE. The phosphate density peak at the headgroups region had a significant dip in PE density with higher density spikes outside and especially inside the membrane, ethanol showed a similar dip in density at the phosphate peak. Ethanol had a larger tendency to stay inside the POPC membrane while DPPC membranes showed no clear differences.

By visual inspection PET monomer stayed inside the membrane, if started there, and moved rapidly into the headgroups region remaining in the region horizontal to the lipid bilayer for the entire simulation with occasional short movements towards the center of the bilayer (Figs. 1b, 2b). The preferred location at the headgroups was the same distance away from the bilayer center as ethanol. When PET was in water as the simulation started, it entered the bilayer in two POPC simulations and stayed at the headgroups region afterwards. However, in one POPC simulation and in all DPPC simulations it stayed in water phase, but often came to the membrane surface and turned horizontal to the surface. Very minor density dips at the phosphate density peak, similarly to PE simulations, were seen in the two simulations where the molecule entered the membrane.

By visual inspection PE tetramer expressed similar rapid movement in water as the PE monomer, although it had a larger preference to the membrane. It stayed at the center or tail groups region of the membrane avoiding the membrane surface, and the headgroups region. It spent more time parallel to the tails but was still able to turn easily. The molecule entered the membrane in all but one simulation and in all cases it did not exit once inside, showing a larger preference to the membrane than the monomer. A similar dip in density at the phosphate peak was observed as with the other molecules.

**Figure 3:**
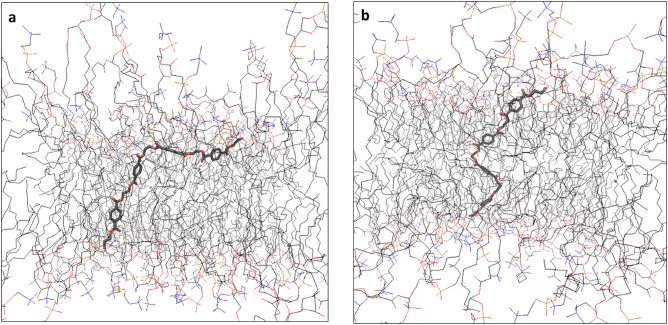
PET-tetramer shapes. (**a**): L-shaped PET-tetramer in POPC bilayer. (**b**): Z-shaped PET-tetramer in POPC-bilayer. Taken as snapshots from the simulations.

By visual inspection PET tetramer stayed inside the membrane every time the simulation started there, unlike the PET monomer. Inside of the membrane PET quickly formed either “L” or “Z” –shapes where two monomer units were able to stay inside the membrane perpendicular to it while the other two would move into the lipid headgroups region and attempt to turn parallel to the membrane. The “L”-shape had one end inside the membrane perpendicular to it and the other end were mostly parallel to it (Fig. 3a). The “Z”-shape had the central inside perpendicularly to the membrane and the terminals almost parallel to the membrane in the headgroups region (Fig. 3b). The length of the tetramer did not allow both terminals to reach parallel position simultaneously. Most of the time monomeric parts of tetramer acted as “anchor points” being parallel to the surface and moved very little compared to the rest of the molecule. When PET tetramers were placed in water before simulation, none of them entered the membrane. The tetramers also tended to fold onto themselves minimizing contact with water, although these folds were not permanent and could unfold after some time. Movement of PET tetramers was overall much slower than the monomers both in water and especially in the membrane. Density dips seen with ethanol and PE at the phosphate density peak were not seen as the molecules did not enter or exit the membrane.

Water pore formation on one membrane leaflet was visually observed in the simulations with PET tetramers when the molecule was located inside the membrane. The formed pores were not permanent, or very pronounced, but were still visible from the standard flux of the membrane surface. Most pronounced pores formed when the molecule was in L-formation or one end moved further away from the membrane center.

Previous simulations of bisphenol A which is structurally similar to PET are in good agreement with our simulation study of PET monomers^11^. Additionally, hexane simulations done by MacCallum and Tieleman showed similar preference in the membrane as our study’s PE monomers and tetramers, which are all simple hydrocarbons^30^.

**Figure 4:**
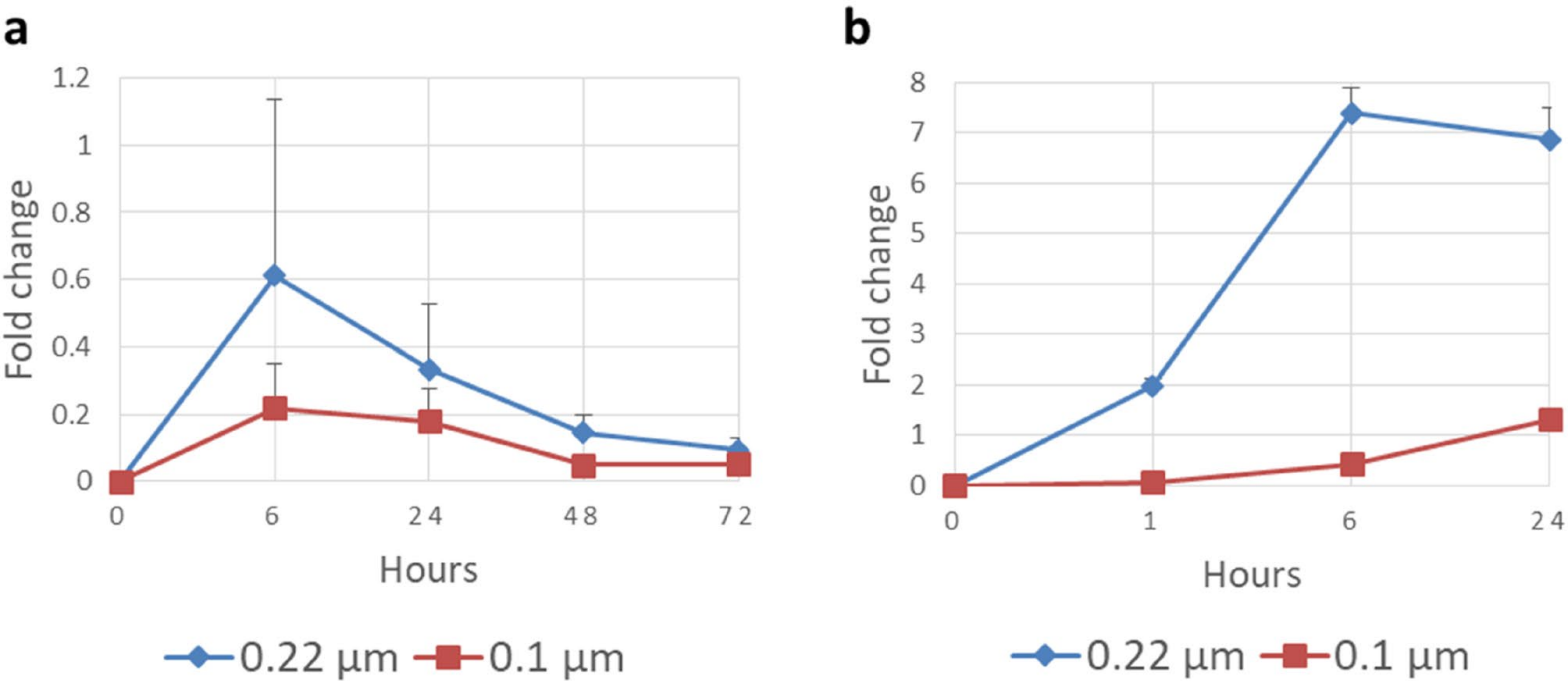
Concentrations of (**a**): PE and (**b**): BHET in the PAMPA experiments presented as a fold change (± SEM) to the integral of the intrinsic standard peaks in NMR.

The size of monomers and tetramers in simulations varied between 0.2 and 5 nm, therefore, they are very small nanoplastics. Nanoparticles of sizes 78 nm and 200 nm have exhibited possible passive transport into cells, while 1 µm sized ones have not^31^. It is important to note, that MPs and nanoplastics can behave somewhat differently^32^. In the experimental permeation study with PAMPA, the permeation of PE and BHET were studied by applying artificial membranes with two pore sizes: 0.1 and 0.22 µm, which fit into the likely upper limits of passive transport. Figure 4 shows that both PE and BHET were able to permeate through the PAMPA membrane.

The permeation speed of PE to the acceptor side decreased over time (Fig. 4). This might be due to the fact that PE particles were seen to adsorb onto the walls of the chamber as well as the donor side of the membrane possibly blocking it over time. This blockage may have caused concentration increases over time on the acceptor side to slow down. The larger non dissolved particles may have blocked the membrane physically. It is also possible that the strong preference to the membrane, as was seen in the simulation studies, could have caused dissolved PE to concentrate into the membrane. PE did have larger concentrations when using the larger pore size membrane, although with a small margin, that did not reach statistical significance at any time points (6 h, p ≈ 0.271; 24 h, p ≈ 0.262; 48 h, p ≈ 0.099; 72 h, p ≈ 0.176). Total cumulative averages of concentrations relative to intrinsic standard concentrations at 72 h were 1.186 for 0.22 µm membrane and 0.501 for 0.1 µm membrane. A small test using the BHET’s NMR-solvents was carried out for 1 h and 6 h timepoints for PE, which showed the same decrease in permeation even between the earlier timepoints. This could point out to the same heavy preference to the membrane as was seen in the simulations with all molecules, but especially for the longer PE tetramer. It is important to note, however, that PE used in PAMPA was a polymer with average particle size between 40 and 48 µm and so was much larger than the monomers and tetramers used in simulations, and thus the plastic was more hydrophobic causing an even stronger preference to the membrane in the PAMPA experiments. For PET, the monomer was the same in both simulations and PAMPA.

BHET concentration increased between 0 and 24 h, except the increase was ended after 6 h with the 0.22 µm membrane (Fig. 4). The 0.22 µm pore membrane had significantly larger concentrations of BHET on acceptor side (1 h, p ≈ 0.0028; 6 h, p ≈ 0.0027; 24 h, p ≈ 0.037) than with 0.1 µm membrane at all time points. A blocking effect similar to PE was observed with BHET although at a smaller scale which may have caused the slow decline between 6 and 24 h timepoints. Total cumulative averages of concentrations relative to intrinsic standard concentrations at 24 h were 16.235 for 0.22 µm membrane and 1.799 for 0.1 µm membrane.

The NMR-solvent used for PE-plastic was not applicable for BHET due to its weaker solubility in water. A possible source of inaccuracy was the variability of viscosity of the PAMPA-reagent between measurement days, which may have also caused the large error at the 6 h timepoint for PE due to worse spread of the reagent on the membrane in one case.

In conclusion PAMPA showed that PE plastic and PET-monomers were able to pass through the artificial membrane and in the simulations of PE monomers, tetramers and PET monomers passive transport across the membrane was also observed. Longer PET chains are more challenging to test using PAMPA due to their poor solubility in PAMPA-compatible solvents and they also did not pass through the membranes in our simulations although they did have the most pronounced effects on its structure. More understanding is needed into how different types of plastic oligomers differ from the ones studied and whether larger amounts of plastics oligomers or longer chains in simulations could have effects on the membrane properties or if they could show aggregation behavior. Other types of experimental methods, in addition to PAMPA, could be explored; especially those ones that could show active transport, such as cell studies, as well as molecular modeling for active transport. More research is required to figure out the best methods for type of transport of plastics.

## Supplementary Information

Supplementary Figures.
