# Supplementary material for: PE and PET oligomers’ interplay with membrane bilayers
Source: Sci Rep. 2022 Feb 9;12:2234. doi: 10.1038/s41598-022-06217-4 (PMC8828856; doi:10.1038/s41598-022-06217-4)
Supplement: Supplementary file 1 — Supplementary Figures. [file 41598_2022_6217_MOESM1_ESM.pdf]

Supplementary information

PE and PET oligomers' interplay with membrane bilayers

Joni Järvenpää, Milla Perkkiö, Riikka Laitinen, Maija Lahtela-Kakkonen

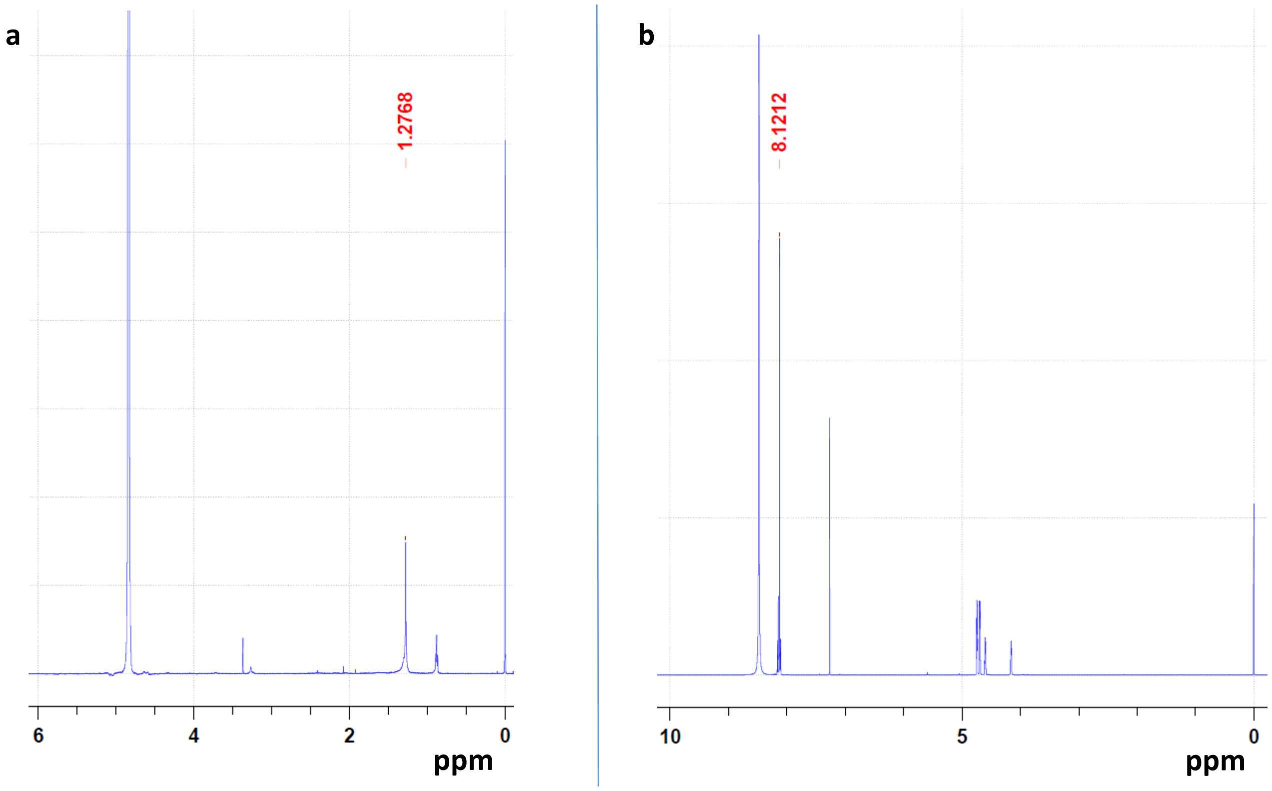

Supplementary Figure S1. NMR-spectra. (**a**): PE NMR-spectra, methylene hydrogen peak indicated. (**b**): BHET NMR-spectra, aromatic hydrogen peak indicated.

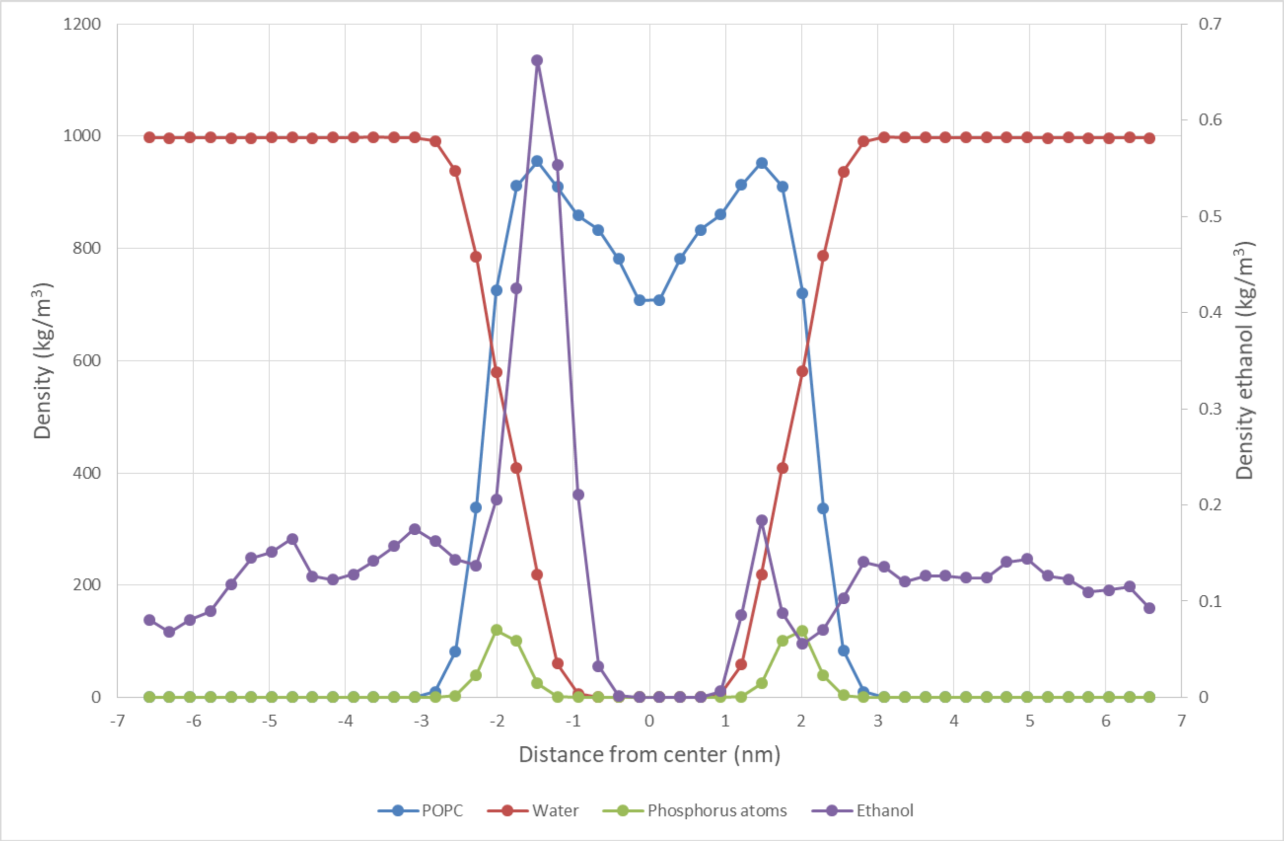

Supplementary Figure S2 Mass density profile of ethanol simulation outside POPC membrane shows that ethanol prefers to locate in the headgroups region of membrane, although with significant time spent in water phase.

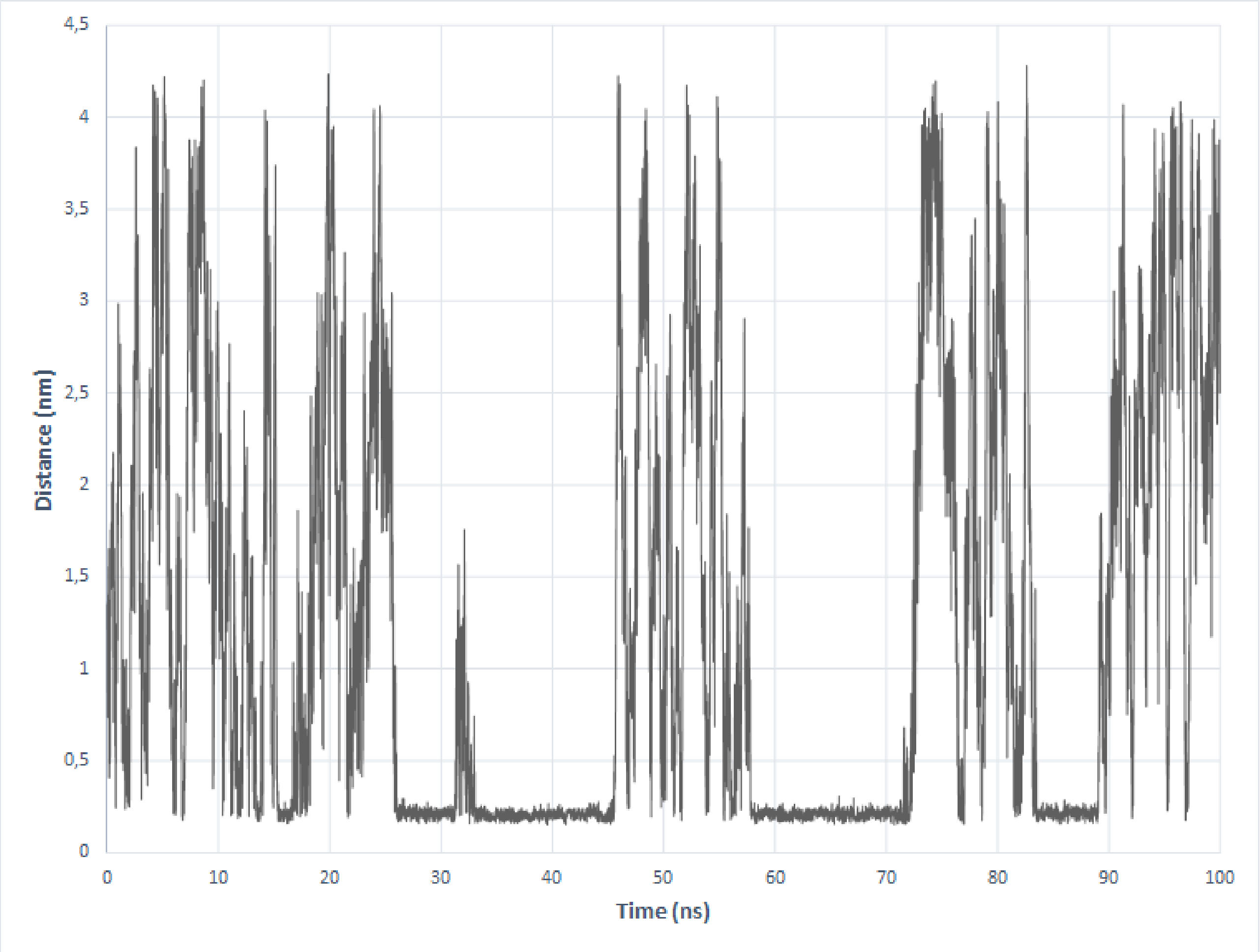

Supplementary Figure S3. The distance of ethanol from the POPC membrane atoms plotted with respect to simulation time in a single selected example simulation run. Figure shows random movement in water is slightly preferred, however the molecule was able to enter the membrane multiple times for short periods at 25 ns, 35 ns, 57 ns and 84 ns.
